# Copper-linked metabolic stress as a potential contributor to gastric epithelial transformation and metastasis

**DOI:** 10.1097/MD.0000000000046590

**Published:** 2025-12-26

**Authors:** Kewei Bi, Xuge Wei, Chao Han, Ning Li, Baishi Wang, Fu Ren

**Affiliations:** a Liaoning Province Key Laboratory for Phenomics of Human Ethnic Specificity and Critical Illness (LPKL-PHESCI), Shenyang Medical College, Shenyang, China; b Shenyang Key Laboratory for Phenomics, Shenyang Medical College, Shenyang, China; c College of Basic Medicine, Shenyang Medical College, Shenyang, China; d Department of Obstetrics and Gynecology, The First Affiliated Hospital of Naval Medical University (Second Military Medical University), Shanghai, China.

**Keywords:** copper, epithelial transition phenotype, gastric tumors, immune evasion, Mendelian randomization, metabolism toxicity stress, single-cell sequencing

## Abstract

Gastric cancer remains a major health burden, and the molecular events that accompany the transition from normal gastric epithelium to malignancy (and the factors that facilitate dissemination) are incompletely defined. In particular, the cellular phenotypes that precede overt transformation and the role of copper-linked metabolic stress in this process are not well understood. We integrated 2 public single-cell RNA-seq datasets of gastric tissues (gene expression omnibus) using canonical correlation analysis-based batch correction and performed quality control and pre/post-integration assessments. Epithelial subclusters were annotated and analyzed by dimensionality reduction, pseudotime inference (monocle; natural-spline model ~sm.ns[Pseudotime]), gene ontology/Kyoto encyclopedia of genes and genomes enrichment, and pathway activity scoring (gene set variation analysis/gene set enrichment analysis). Immune dysfunction/exclusion was evaluated (e.g., tumor immune dysfunction and exclusion). To evaluate genetic evidence, we conducted two-sample Mendelian randomization (MR) using circulating copper as the exposure and benign gastric tumors (FinnGen) and gastric cancer (Genome-Wide Association Study Catalog) as outcomes; standard diagnostics (e.g., *F*-statistics, I²_GX, pleiotropy/heterogeneity tests, MR-PRESSO) were applied. We identified a putative transitional epithelial phenotype between normal and malignant states, characterized by attenuation of lineage functions (e.g., gastric-acid-related signatures) and enrichment of stress-adaptation programs. Along pseudotime, module scores for stress response to copper ion increased from normal to transition cells and decreased along the tumor branch; metastatic epithelial cells showed attenuated copper-stress signatures together with enhanced motility/tumor programs, consistent with phenotypic convergence across metastatic sites. Primary tumor epithelium exhibited reinforced cell-cycle activity and immune-response attenuation, with immune dysfunction exceeding that of normal epithelium. In MR analyses, genetically proxied variation in circulating copper was positively associated with risk of both benign and malignant gastric tumors, with directions concordant across estimators and diagnostics not indicating major violations (findings consistent with a deleterious causal contribution under standard MR assumptions). Single-cell and genetic evidence prioritize copper-linked metabolic stress and epithelial remodeling (together with immune dysfunction) as testable axes in early gastric epithelial transformation and dissemination. While the patterns are consistent with a causal contribution of higher systemic copper, they do not constitute proof; orthogonal tissue validation and functional perturbation of copper-handling/cuproptosis pathways, alongside expanded genetic analyses (e.g., multivariable/colocalization), are warranted. These insights suggest potential avenues for prevention and targeted intervention.

## 1. Introduction

Throughout history, stomach cancer has remained one of the major challenges to human health and well-being, marked by its high lethality, with a 5-year survival rate of only around 20%.^[1,2]^ Many studies have revealed various characteristics of tumor cells, such as drug resistance, uncontrolled proliferation, and enhanced migratory ability.^[3–5]^ However, the phenotypic alterations of normal tissue cells prior to tumor initiation remain poorly understood. A comprehensive characterization of these changes, together with the accompanying shifts in gene expression and cell phenotypes throughout carcinogenesis, is critical for elucidating the fundamental mechanisms and key factors that drive cellular transformation. Gastric cancer is highly prone to metastasis and is frequently diagnosed at an advanced stage, with >50% of patients presenting with metastatic disease at the time of diagnosis.^[6]^ Therefore, comparative analyses of gene expression profiles between gastric tumor cells in situ and those in metastatic lesions may provide further insights into the mechanisms underlying tumorigenesis and metastasis. Previous studies have demonstrated that gastric tumor cells selectively colonize microenvironments in the peritoneum that are favorable for their growth.^[7–9]^ These observations suggest that tumor cells can actively adapt to, and survive in, metastatic niches.^[10–12]^ Taken together, they raise the possibility that some factors may not only predispose cells to primary tumorigenesis but also facilitate progression toward metastasis. In this context, our data point to copper-linked cytotoxic stress as a putative pro-carcinogenic influence. In single-cell transcriptomes spanning normal gastric epithelium, primary tumors, and metastases, metastatic cells exhibited attenuated copper-stress signatures, whereas a subset of epithelial cells between normal and malignant states showed pronounced copper-toxicity-related features. Complementing these observations, Mendelian randomization (MR) analyses provided tentative evidence, under standard MR assumptions, consistent with a deleterious contribution of higher genetically proxied variation in circulating copper to gastric cancer risk.

## 2. Materials and methods

### 2.1. Single-cell transcriptome sequencing data

This study utilized 2 sets of 10× Genomics single-cell transcriptome sequencing data from the gene expression omnibus public database: GSE184198 and GSE163558.

### 2.2. Single-cell sequencing data processing

After filtering and integration of 2 sets of single-cell sequencing data using the R package Seurat (Satija Lab, New York Genome Center, New York), a total of 59,462 cells were obtained. To mitigate potential batch effects across datasets, Seurat canonical correlation analysis-based anchor integration was used. Raw counts were normalized and variable features identified for each dataset. Integration features were then selected and cross-dataset anchors computed via canonical correlation analysis, followed by data integration. We utilized 2 dimensionality reduction methods, Uniform Manifold Approximation and Projection (UMAP) and t-distributed stochastic neighbor embedding, to visualize single-cell gene expression features in two-dimensional space. Subsequently, using the R package SingleR and the expression of characteristic marker genes from different cell types, we evaluated and identified cell types, resulting in a total of 8 cell types, among of which, 2932 epithelial cells in total.

### 2.3. Processing of epithelial cell gene expression data

From the clustering results of all cells, epithelial cell were isolated. Subsequently, dimensionality reduction clustering was applied to the epithelial cells. The characteristic genes of each epithelial cell subcluster were identified using the FindAllMarkers function in the Seurat R package.

### 2.4. Pseudo-temporal analysis of gene expression in the epithelial cells

Pseudo-temporal analysis of gene expression in epithelial cells was conducted using the monocle R package (Trapnell Lab, Department of Genome Sciences, University of Washington, Seattle).

### 2.5. Gene ontology (GO) and Kyoto encyclopedia of genes and genomes (KEGG) signaling pathways enrichment analysis

Utilizing the Seurat R package, analysis was conducted on the characteristic genes of each epithelial cell subgroup, along with the differentially expressed genes (DEGs) between different subclusters. The selection criteria for significant characteristic genes were set at avg_log2FC >1 and p_val_adj <.05. Employing the characteristic genes of each epithelial cell cluster and the DEGs between 2 clusters, GO and KEGG signaling pathway enrichment analyses were performed using the R packages GOplot and clusterProfiler.

### 2.6. Gene set enrichment analysis (GSEA) and gene set variation analysis (GSVA)

Gene expression analysis was conducted between pairwise distinct epithelial cell clusters to obtain information on differential gene expression. Subsequently, GSEA and GSVA analyses were performed using the R packages clusterProfiler and GSVA, respectively, with the HALLMARK gene set as the targeted enrichment gene sets.

### 2.7. Differential analysis of immune-related cells and functions

The gene set file for immune-related cells and functions was downloaded from the GSEA website (https://www.gsea-msigdb.org/gsea/index.jsp), organized into an immune.gmt file, and used to compute scores for different epithelial cell clusters in terms of immune-related cells and functions. The R package GSVA was employed to calculate scores for different epithelial cell subgroups in terms of immune-related cells and functions, and the Wilcoxon test was used to assess score differences between different groups.

### 2.8. Differential analysis of immune checkpoint-related genes

Through literature review, immune checkpoint-related genes were collected and integrated, then organized into the ImmuneCheckpointGene.txt file (File S1, Supplemental Digital Content, http://links.lww.com/MD/R20). The differential expression of immune checkpoint-related genes between different groups was assessed using the Wilcoxon test.

### 2.9. Differential analysis of tumor immune dysfunction and exclusion (TIDE) scores

Utilizing the website (http://tide.dfci.harvard.edu/) for computing TIDE scores of different epithelial cell subgroups, the Wilcoxon test was employed to analyze differences in TIDE scores among distinct subclusters of epithelial cell.

### 2.10. MR analysis

*Exposure data source*: The Genome-Wide Association Study (GWAS) data for blood copper levels were obtained from the IEU Open GWAS Project database, specifically from dataset ieu-a-1073. *Outcome data*: GWAS data for benign gastric tumors were sourced from the FinnGen database (dataset: finngen_R10_CD2_BENIGN_STOMACH), and data for malignant gastric tumors were obtained from the GWAS Catalog (dataset: GCST90041806). The R package TwoSampleMR was utilized to perform the selection of instrumental variable SNPs (single nucleotide polymorphisms), as well as the Mendelian randomization analysis and related testing procedures. SNPs associated with the exposure were filtered based on *P*-value thresholds (*P* < 5 × 10^‐6^) and linkage disequilibrium criteria (using the default parameters of the TwoSampleMR package, MRC Integrative Epidemiology Unit, University of Bristol, Bristol, UK). Consequently, 6 SNPs were identified as instrumental variables for the exposure factor.

## 3. Results

### 3.1. The cellular landscape in the tumor microenvironment of gastric cancer

**Figure 1.**
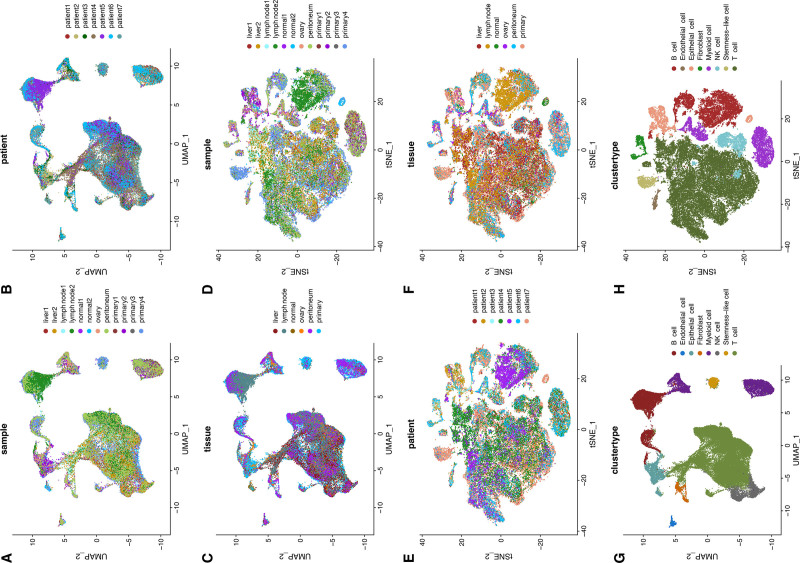
Distinct cell clusters identified in primary and metastatic gastric cancer tissues through dimensionality reduction of gene expression profiles and cell-type annotation. (A–C) UMAP visualization showing the origins of cells, patients, and tissues. (D–F) t-SNE visualization showing the origins of cells, patients, and tissues. (G and H) Distribution of different cell types on UMAP and t-SNE plots. t-SNE = t-distributed stochastic neighbor embedding, UMAP = Uniform Manifold Approximation and Projection.

To mitigate inter-study batch effects and to characterize cellular phenotypic remodeling during the carcinogenesis and metastasis of gastric cancer, we integrated 2 gene expression omnibus scRNA-seq datasets (GSE184198 and GSE163558). After batch correction, UMAP and t-distributed stochastic neighbor embeddings displayed clear subpopulation structure with substantially reduced batch-associated variation, resulting in a more even distribution of cells across samples, patients, and tissues (Fig. 1A–F and Fig. S1, Supplemental Digital Content, https://links.lww.com/MD/R18). Subsequently, cell subpopulations were identified and characterized using the SingleR algorithm with reference expression profiles of specific cell type markers. Eight distinct cell subclusters were identified, comprising T cells, B cells, endothelial cells, epithelial cells, fibroblasts, myeloid cells, NK cells, and stem-like cells. Notably, their marker genes exhibited characteristic expression patterns across subclusters (Fig. 1G and H, and Fig. S2, Supplemental Digital Content, https://links.lww.com/MD/R18).

### 3.2. Dynamic emergence of transitional phenotype cells in the tumorigenic evolution of gastric epithelial cells

**Figure 2.**
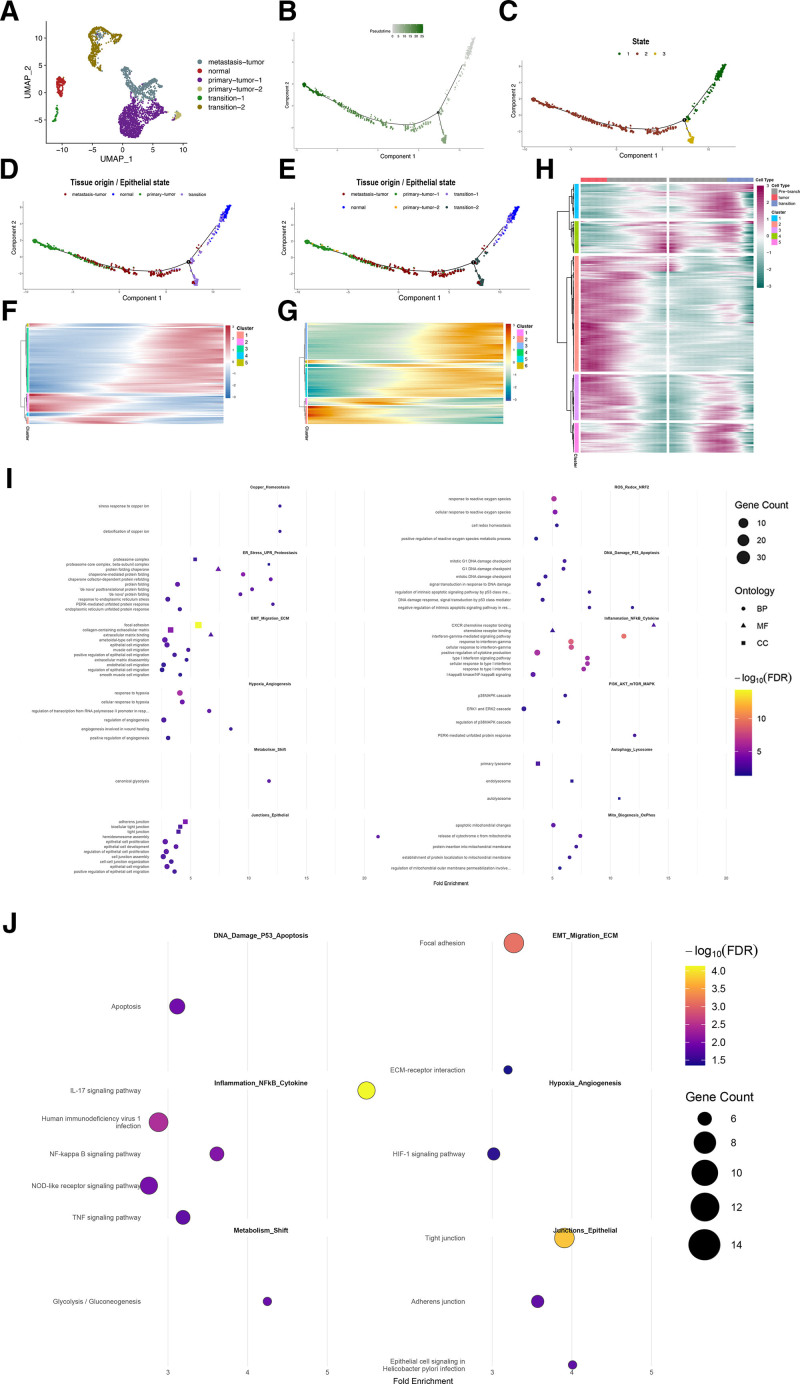
Dimensionality reduction-based clustering analysis, pseudo-temporal profiling analysis, and heatmaps of epithelial cell gene expression. (A) The representation of epithelial cells of various subtypes in UMAP. (B–E) The pseudo-temporal profile of gene expression in epithelial cells, labeled respectively as pseudotime progression, state, and tissue origin/ epithelial state. (F–H) The expression heatmaps of genes associated with pseudo-temporal patterns, (F) heatmap of state-associated genes; (G) heatmap of peudotime-associated genes; (H) heatmap of branch-associated genes. (I) GO enrichment of pseudotime-associated epithelial genes. Bubble size encodes gene count; color encodes (log10[FDR]; the *x*-axis shows fold enrichment). Multiple testing was controlled by Benjamini–Hochberg FDR. (J) KEGG pathway enrichment of pseudotime-associated epithelial genes. color encodes (log10[FDR]; the *x*-axis shows fold enrichment). Multiple testing was controlled by Benjamini–Hochberg FDR. GO = gene ontology, KEGG = Kyoto encyclopedia of genes and genomes, UMAP = Uniform Manifold Approximation and Projection.

Discrete clusters of epithelial cells were identified within the heterogeneous cell populations of the gastric cancer microenvironment using dimensionality reduction and cell-type annotation. Notably, transitional phenotype cells were observed to emerge dynamically during gastric epithelial carcinogenesis, suggesting a potential role in tumor evolution. Subsequently, UMAP was applied to visualize the data in two-dimensional space, and 6 distinct epithelial cell phenotypes were characterized. By integrating epithelial cell tissue origins with marker gene expression profiles across clusters, we identified the distinction between malignant and non-tumor epithelial cells (Fig. 2A and Fig. S3, Supplemental Digital Content, https://links.lww.com/MD/R18). Notably, nonmalignant epithelial subtypes were not confined to a single subcluster but rather dispersed across 3 subclusters located on the left side of the UMAP plot. Joint analysis of malignant epithelial marker expression and tissue origins revealed that the far left cluster corresponds to the normal epithelial cell subcluster. Simultaneously, on the left side of the plot, besides the centrally positioned normal epithelial cells, 2 additional epithelial subtypes (situated above and below) are also classified as non-tumor cell subclusters. These 2 clusters are provisionally designated as “transition” subclusters, with “transition” denoting an intermediate cellular phenotype in the progression from normal to malignant states (Fig. 2A). Pseudotime analysis of epithelial subclusters revealed coherent yet divergent temporal programs across subgroups. In the 2-D trajectory, pseudotime initiates in the upper-right region and proceeds leftward, with a clear bifurcation in the mid-trajectory (Fig. 2B and C). When cells were colored by tissue origin and epithelial state, the trajectory appeared to originate predominantly from normal and transition epithelial states; along the path, a minor downward branch comprised mainly transition cells with limited metastatic infiltration, whereas a larger leftward branch was enriched for metastatic and in situ tumor epithelial cells (Fig. 2D and E). Coloring by finer delineation of tissue origin and epithelial state further indicated that the start of the trajectory contains mostly normal and transition-1 cells, with transition-1 mapping slightly downstream of normal, consistent with an early shift from normal toward transitional programs (Fig. 2E). At the branch point, the downward arm was dominated by transition-2 cells with a small metastasis component, whereas the leftward arm was composed largely of metastatic and primary-tumor-like states, suggesting a stepwise progression from normal to transition-1 before reaching a critical bifurcation. Along the inferred timeline, metastasis-associated states tended to appear earlier than primary-tumor-like states, indicating earlier activation of dissemination-related programs rather than a strict chronological precedence (Fig. 2D and E). To summarize dynamic programs along pseudotime, we clustered genes by their temporal profiles and visualized scaled expression as heatmaps ordered by pseudotime (gene modules 1–5 in Fig. 2F; 1–6 in Fig. 2G). The modules segregate into 3 canonical patterns: monotonic up-regulation, monotonic down-regulation, and transient (biphasic) responses. As pseudotime increases, subsets of genes show sustained induction within specific epithelial subgroups, whereas others are progressively repressed; a third set displays early induction followed by attenuation, consistent with transient activation of intermediate programs (Fig. 2F and G). Significant genes are listed in Lists S1 and S2, Supplemental Digital Content, https://links.lww.com/MD/R19. These patterns recapitulate the heterogeneous kinetics observed across epithelial states and highlight coordinated yet divergent transcriptional trajectories along pseudotime. Using branched expression analysis modeling, we identified gene modules with branch-dependent temporal profiles along the pseudotime trajectory. When genes were ordered by pseudotime and grouped into modules, 3 recurrent patterns emerged: monotonic induction, monotonic repression, and transient (biphasic) responses. Notably, a subset of branch-point-associated genes showed early up-regulation followed by attenuation after the bifurcation, whereas others exhibited sustained, fate-specific induction along the tumor-directed versus transition-directed arms (Fig. 2H). Significant genes are listed in List S3, Supplemental Digital Content, https://links.lww.com/MD/R19. Pseudotime-associated DEGs (identified by a natural-spline model, ~sm.ns[Pseudotime]) showed coherent enrichment of pathways expected under copper stress and epithelial state transitions. In GO enrichment, we observed significant terms for copper homeostasis (e.g., “detoxification of copper ion” and “stress response to copper ion”), ROS_Redox_NRF2 (Reactive Oxygen Species, Redox Homeostasis, and NRF2 Pathway, e.g., response to reactive oxygen species, positive regulation of reactive oxygen species metabolic process, and cell redox homeostasis), ER-stress/UPR (including PERK-mediated UPR), p53-linked DNA-damage and apoptosis, and mitochondrial apoptotic processes (e.g., cytochrome-c release), together with epithelial junction organization, ECM/focal-adhesion remodeling, cell migration, hypoxia/angiogenesis, and inflammatory signaling (NF-κB/interferon) (Fig. 2I). KEGG analysis converged on related themes, including ECM–receptor interaction, focal adhesion, adherens/tight junction pathways, HIF-1 signaling, apoptosis, and immune/inflammatory pathways (NF-κB, NOD-like receptor, IL-17), alongside metabolic reprogramming (glycolysis/gluconeogenesis) (Fig. 2J). Collectively, these enrichments are consistent with copper-toxicity and copper-metabolism-linked stress responses, and with epithelial remodeling processes that align with a normal → transition → tumor-like progression inferred from the pseudotime analysis (while not by themselves constituting proof of chronology).

### 3.3. The metabolic stress induced by toxic copper ions promotes the carcinogenesis of normal gastric cells

**Figure 3.**
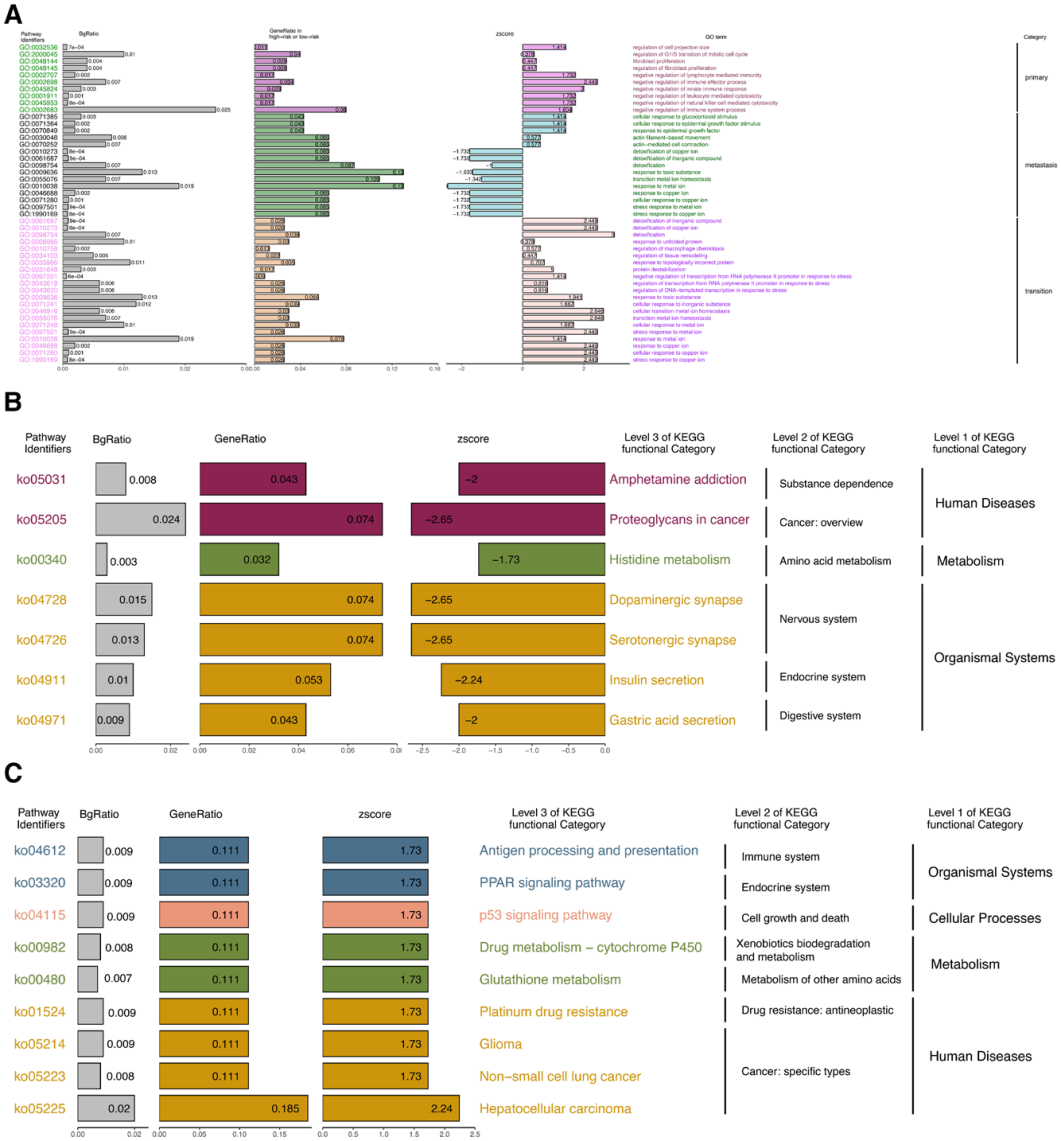
GO and KEGG enrichment analysis. (A) Enrichment analysis of GO and KEGG signaling pathways using characteristic genes from various cellular subgroups. (B) The enrichment analysis of KEGG signaling pathways for downregulated genes in epithelial cells of the transition subtype compared to normal epithelial cells. (C) The enrichment analysis of KEGG signaling pathways for upregulated genes in metastatic gastric cancer cells compared to in situ tumor cells. GO = gene ontology, KEGG = Kyoto encyclopedia of genes and genomes.

In primary epithelial tumor cells, GO enrichment of phenotype-specific signatures indicates 3 prominent features: progressive activation of cell-cycle and proliferative programs, consistent with intensification during in situ carcinogenesis; increased cell-projection/structural organization; and attenuated immune-response activity, exemplified by the enrichment of terms such as *negative regulation of lymphocyte-mediated immunity*, *negative regulation of innate immune response*, and *negative regulation of immune system process* (Fig. 3A). These patterns suggest that immune evasion may begin to emerge within the primary niche.

In the transition phenotype (represented by the transition-2 subcluster), enrichment results point to a marked response to toxic metal-induced metabolic stress, with copper-linked terms among the most salient, including *cellular transition metal ion homeostasis*, *detoxification of copper ion*, *response to copper ion*, and *stress response to copper ion* (Fig. 3A). Additionally, we computed per-cell module scores for the GO term “stress response to copper ion” and projected them onto the epithelial pseudotime trajectory. Along pseudotime, *stress response to copper ion* scores increase from normal to transition epithelial cells and decrease on the tumor branch, indicating that copper-linked stress is most prominent in the transition phenotype. This is consistent with, but does not by itself prove, a role for copper-induced metabolic stress in the early steps of gastric epithelial transformation (Fig. S4, Supplemental Digital Content, https://links.lww.com/MD/R18). Additional enrichments related to transcriptional stress and proteostasis (e.g., *regulation of transcription from RNA polymerase II promoter in response to stress*, *response to unfolded protein*, and *protein destabilization*) are consistent with broader cellular stress loads. Functionally, the transition phenotype also shows diminished baseline gastric functions, including reduced gastric-acid secretion relative to normal epithelium (Fig. 3B). By contrast, metastatic epithelial cells display the opposite pattern for several copper-related processes (e.g., negative enrichment for *detoxification of copper ion* and *response to copper ion*) alongside higher activity of growth- and motility-associated programs, including *cellular response to epidermal growth factor stimulus*, *response to epidermal growth factor*, and *actin filament-based movement* (Fig. 3A). KEGG analyses further indicate broad activation of cancer-featured signaling pathways (e.g., *glioma*, *non-small cell lung cancer*, *hepatocellular carcinoma*) and enhanced drug-resistance signatures in metastatic cells (Fig. 3C). Together, these enrichment patterns are consistent with a progression from normal epithelium to a copper-stress-responsive transition state and onward to primary and metastatic tumor phenotypes, while not, by themselves, establishing real-time chronological order.

### 3.4. The gastric cancer cells manifest an active cellular proliferation phenotype

**Figure 4.**
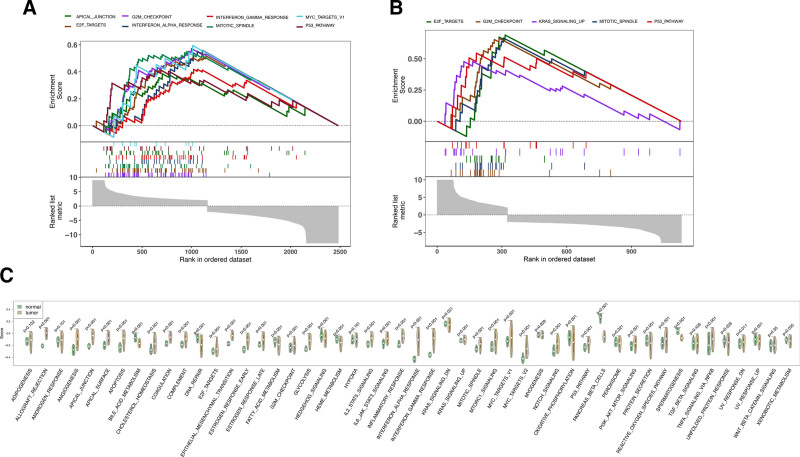
Conducting gene set enrichment analysis (GSEA) and gene set variation analysis (GSVA) on different phenotypic subclusters of epithelial cells. (A) GSEA analysis using differentially expressed genes (primary vs transition) between primary and transition phenotypic epithelial cells. (B) GSEA analysis using differentially expressed genes (metastasis vs transition) between metastasis and transition phenotypic epithelial cells. (C) GSVA using differentially expressed genes between tumor and normal epithelial cells.

The preceding study findings suggest that transition cells present an intermediate cellular phenotype during the carcinogenic transformation of normal gastric epithelial cells. Hence, contrasting transition cells with normal epithelial cells will facilitate the elucidation of phenotypic alterations in tumor cells. GSEA conducted on primary and metastatic tumor cells compared to transition cells reveals a significant activation of the cell cycle process in both primary and metastatic tumor cells. This is evident from the enrichment of genes associated with the G2/M checkpoint and spindle-related genes during cell division (Fig. 4A and B). Additionally, GSEA results identified the functional gene sets contributing to the proliferative phenotype of primary and metastatic tumor cells. Notably, primary and metastatic tumors show significant enhancement in gene sets such as “E2F_TARGETS” and ‘P53_PATHWAY’, implying the mechanism driving the cell cycle process. Moreover, primary tumor cells exhibit upregulation of the MYC signaling pathway and response to alpha and beta interferon signaling pathways, distinguishing them to some extent from metastatic tumor cells that predominantly utilize the KRAS signaling pathway, which reveals the distinct pathway adopting by primary and metastatic tumor cells (Fig. 4A and B). Furthermore, to uncover the metabolic characteristics of tumor cells, primary and metastatic tumor cells are compared with normal epithelial cells using GSVA, revealing enhanced metabolic phenotypes across various aspects including cell proliferation metabolism (“G2M_CHECKPOINT,” “MITOTIC_SPINDLE”), cellular microenvironment (“ANGIOGENESIS,” “APICAL_JUNCTION,” “APICAL_SURFACE”), and cellular substance metabolism (‘GLYCOLYSIS’) (Fig. 4C). Notably, “EPITHELIAL_MESENCHYMAL_TRANSITION” and weakened DNA repair capabilities compared to normal gastric epithelial cells. Furthermore, tumor cells show diminished activity in certain functionally active gene sets in gastric epithelial cells, such as ‘PROTEIN_SECRETION’ (Fig. 4C), providing robust evidence for the process of carcinogenic transformation of normal gastric epithelial cells.

### 3.5. Gastric cancer cells achieve immune evasion by mediating immune-related functional dysregulation through abnormal expression of immune checkpoint-related genes

**Figure 5.**
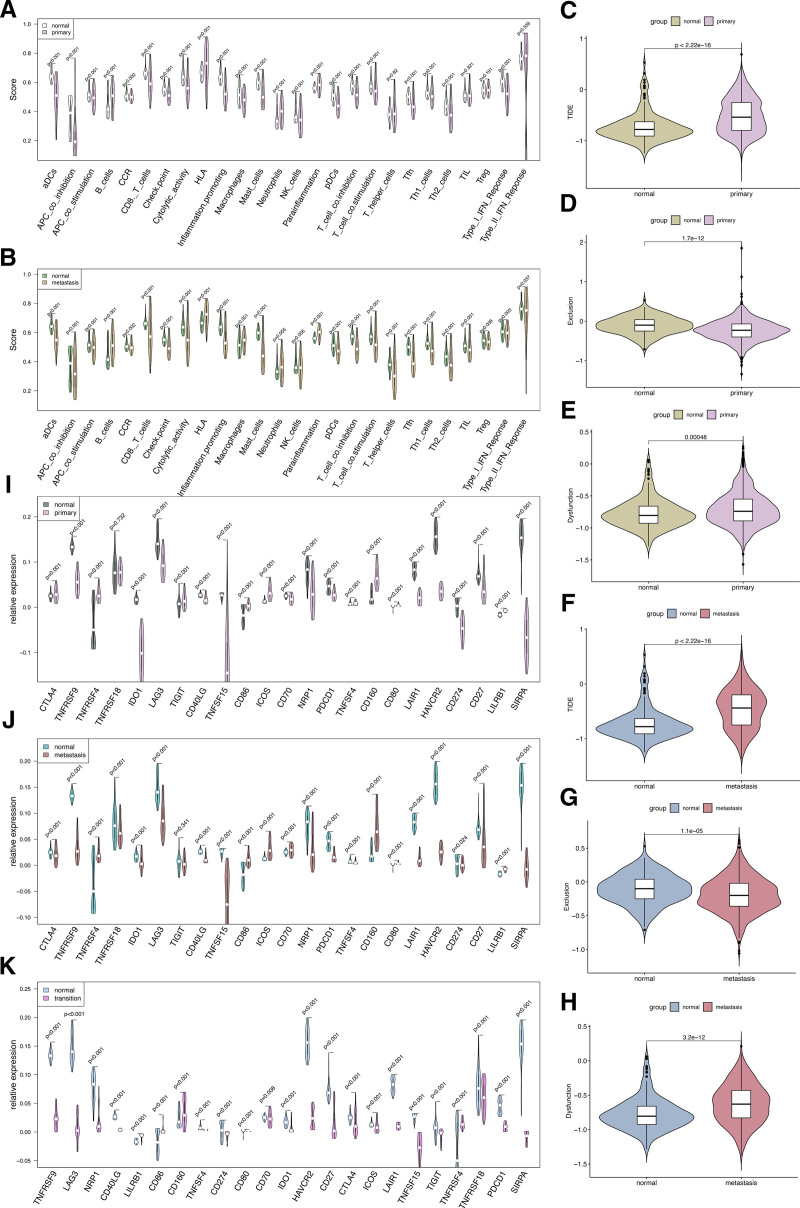
Differential expression analysis of immune checkpoint-related genes among various subtypes of epithelial cells, in conjunction with GSVA utilizing gene sets associated with immune functions, and TIDE analysis. (A and B) The GSVA of immune-related functionalities among different phenotypic subgroups of epithelial cells. (C–H) The TIDE analysis between different phenotypic gastric epithelial cells. (C) The difference in TIDE scores between primary gastric tumor cells and normal gastric epithelial cells; (D) the difference in exclusion scores between primary gastric tumor cells and normal gastric epithelial cells; (E) the difference in dysfunction scores between primary gastric tumor cells and normal gastric epithelial cells; (F) the difference in TIDE scores between metastatic gastric tumor cells and normal gastric epithelial cells; (G) the difference in exclusion scores between metastatic gastric tumor cells and normal gastric epithelial cells; (H) the difference in dysfunction scores between metastatic gastric tumor cells and normal gastric epithelial cells. (I–K) Differential expression of immune checkpoint-related genes across various phenotypic subclusters, including comparisons between primary vs normal, metastasis vs normal, and transition vs normal gastric epithelial cells. GSVA = gene set variation analysis, TIDE = tumor immune dysfunction and exclusion.

Based on the previous research findings, normal gastric epithelial cells undergo multiple stages, gradually evolving into tumor cells with proliferating phenotype, involving imbalanced cellular proliferation metabolism due to toxic stress such as copper ions. Dysregulations of immune-related functions also play significant roles in the formation of gastric cancer tumors. Utilizing GSVA, significant differences were observed in immune-related functions and cells between primary and metastatic tumor cells compared to normal gastric epithelial cells (Fig. 5A and B), endowing tumor cells with immune escape potential. Significantly, the TIDE scores of primary and metastatic gastric cancer cells were markedly higher than that of normal gastric epithelial cells, indicating a certain degree of immune escape potential in primary and metastatic gastric cancer cells (Fig. 5C and F). It is known that tumor immune escape may occur through 2 possible mechanisms. Firstly, by impeding immune cell access to tumor cells, thereby reducing immune cell infiltration into tumor niche and weakening the cytotoxic effects on tumor cells. However, in this study, neither primary nor metastatic gastric cancer cells adopted this approach (Fig. 5D and G). Therefore, the second mechanism for tumor immune escape involves the dysregulation of immune-related functions. Evaluation of immune-related function dysregulation in primary and metastatic gastric tumor cells revealed their potential to interfere with immune cytotoxicity to achieve immune escape (Fig. 5E and H). As immune checkpoint-related genes play crucial roles in recognition and activation of normal immune cytotoxic functions, further differential analysis of immune checkpoint-related gene expression is warranted. The results showed significant differences in the expression of multiple immune checkpoint-related genes between primary and metastatic gastric cancer cells compared to normal gastric epithelial cells, even evident in epithelial cells with transition phenotype before tumor formation (Fig. 5I–K). These findings seem to suggest specific abnormal expression of immune checkpoint-related genes in gastric cancer cells disrupt normal immune functions of immune cells and thereby achieving immune escape.

### 3.6. MR indicates a potential causal contribution of circulating copper to gastric cancer

**Figure 6.**
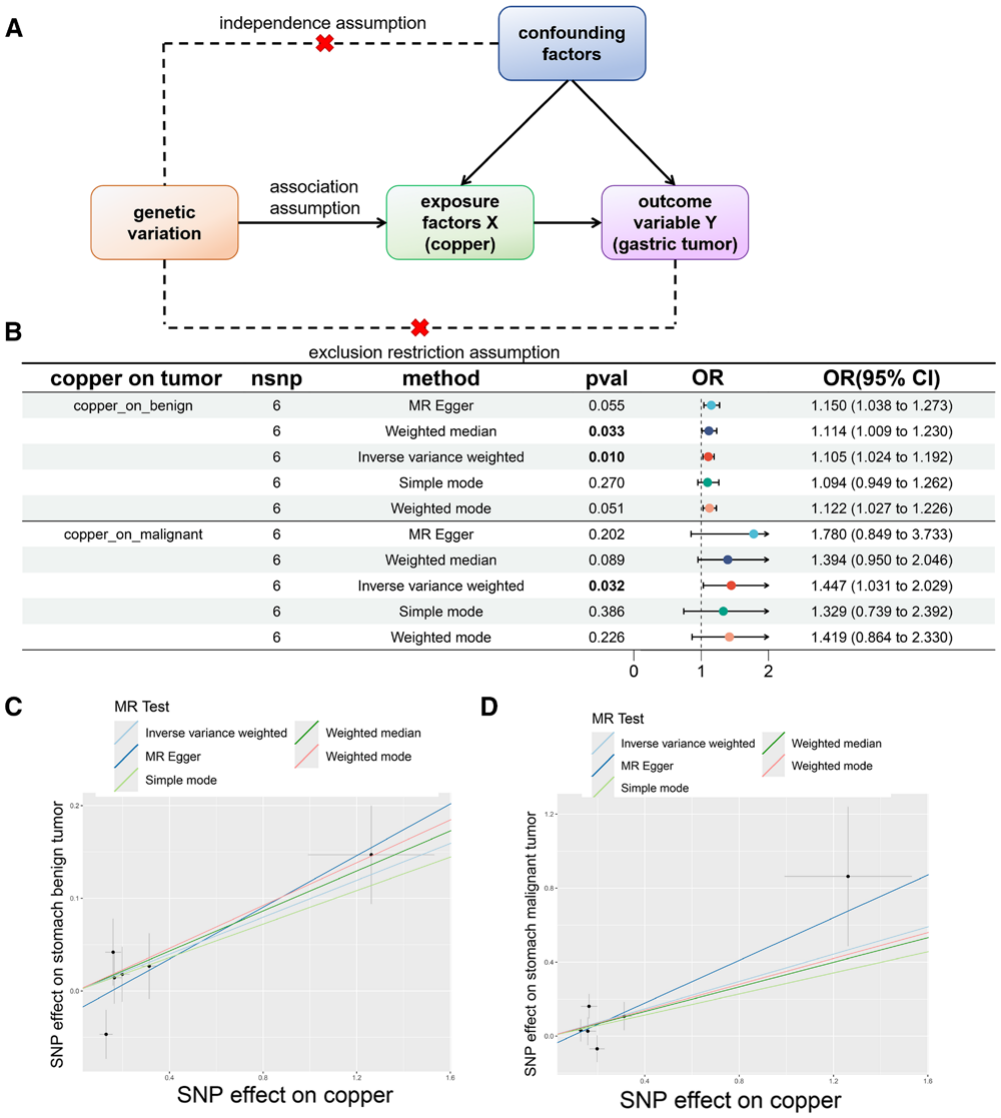
Mendelian randomization analysis of the causal relationship between blood copper levels and gastric tumors. (A) Conceptualization of a Mendelian randomization analysis of the causal relationship between blood copper levels and gastric tumors. (B) Forest plot presenting the results of the causal relationship analysis between blood copper levels and benign and malignant gastric tumors. (C and D) The scatter plots illustrates the effects of instrumental variable SNPs on exposure and outcome variables. Specifically, panel C shows the effects of instrumental variable SNPs on benign gastric tumors, while panel D shows their effects on malignant gastric tumors. SNP = single nucleotide polymorphism.

Prior work suggests that copper-linked metabolic stress may accompany the shift from normal to transitional to malignant gastric epithelial states. To test whether higher systemic copper is consistent with a causal contribution to tumor initiation, we performed two-sample MR using circulating copper as the exposure (IEU OpenGWAS) and benign gastric tumor (FinnGen) and gastric cancer (GWAS Catalog) as outcomes (Fig. 6A). Genome-wide significant copper-associated variants were clumped for linkage disequilibrium , yielding 6 independent SNP instruments (Table 1 and Fig. S5A, B, Supplemental Digital Content, https://links.lww.com/MD/R18). Per-SNP F-statistics were >10, and I²_GX was evaluated to gauge potential regression dilution of MR-Egger (Table 1). We assessed pleiotropy/heterogeneity using Cochran *Q* and the MR-Egger intercept, and ran MR-PRESSO (global/outlier/distortion tests) (Table 2 and Fig. S5C, D, Supplemental Digital Content, https://links.lww.com/MD/R18). These diagnostics did not indicate major violations of MR assumptions; nevertheless, we interpret estimates cautiously given the limited number of instruments (n = 6). Across 5 estimators (IVW, weighted median, MR-Egger, simple mode, weighted mode), effect directions were concordant, with the IVW estimate indicating a positive association between genetically proxied copper and risk of both benign and malignant gastric tumors, and a comparatively larger effect for gastric cancer (Fig. 6B). Leave-one-out analyses showed that removing any single instrument had minimal impact on the pooled estimates (Fig. S5E and F, Supplemental Digital Content, https://links.lww.com/MD/R18). Scatter and funnel plots illustrate the consistency of SNP-level effects and the absence of strong asymmetry (Fig. 6C, D and Fig. S5C and D, Supplemental Digital Content, https://links.lww.com/MD/R18). Taken together, these MR analyses are consistent with higher genetically proxied circulating copper being deleterious for benign and malignant gastric tumor risk under standard MR assumptions, but (given the small instrument set and residual uncertainty about pleiotropy) they do not, by themselves, establish causality.

**Table 1:** Statistical results of SNPs associations with exposure and outcome variables in Mendelian randomization analysis.

	Exposure (copper)							Outcome (benign)				Outcome (malignant)			
SNP	Beta	SE	*P* value	Sample size		Instrument strength (F)	I²GX	Beta	SE	*P* value	Sample size	Beta	SE	*P* value	Sample size
rs10014072	-0.164	0.034	1.13E‐06		2603	23.25	0.98	‐0.014	0.028	.60657	412,181	-0.162	0.066	.014818	456,348
rs1175550	0.198	0.032	5.03E‐10		2603	38.26		0.018	0.030	.54149	412,181	-0.068	0.072	.343799	456,348
rs12153606	-0.159	0.034	2.50E‐06		2603	21.85		‐0.042	0.036	.24597	412,181	-0.027	0.075	.722192	456,348
rs12582659	1.262	0.27	2.86E‐06		2603	21.83		0.147	0.053	.00561	412,181	0.863	0.377	.022135	456,348
rs2769264	0.313	0.034	2.63E‐20		2603	84.68		0.027	0.035	.44726	412,181	0.108	0.077	.159097	456,348
rs3857536	-0.129	0.028	4.08E‐06		2603	21.21		0.047	0.026	.0775	412,181	-0.031	0.060	.603852	456,348

SNP = single nucleotide polymorphism.

**Table 2:** Pleiotropy and heterogeneity tests for instrumental variable SNPs.

	Pleiotropy test						Heterogeneity test					
	MR-Egger			MR-PRESSO			MR-Egger			IVW		
Outcome	Intercept	SE	*P* value	*P* value	Outliers	Distortion p	*Q*	*Q*_df	*Q*_p-value	*Q*	*Q*_df	*Q*_p-value
Benign	‐0.021	0.019	.325	.515	None	NA	4.388	4	0.356	5.769	5	0.329
Malignant	‐0.052	0.083	.563	.268	None	NA	6.863	4	0.143	7.542	5	0.183

SNP = single nucleotide polymorphism.

## 4. Discussion

### 4.1. Overview and objectives

This study set out to characterize epithelial state transitions in gastric cancer at single-cell resolution and to evaluate whether copper-linked metabolic stress aligns with these transitions and with genetic evidence consistent with causality. Specifically, we asked: whether a transition epithelial phenotype can be delineated between normal and malignant states; whether copper-related stress programs are most prominent in this transition state; and whether genetically proxied circulating copper shows associations consistent with a causal contribution to benign and malignant gastric tumor risk under standard MR assumptions.

### 4.2. Cellular composition and epithelial heterogeneity

Unsupervised clustering confirmed that gastric tumors contain diverse cell types. Within epithelium, cells from in situ tumors resolved into 2 subclusters, whereas epithelial cells from metastases across distinct organs converged into a largely single cluster, suggesting phenotypic convergence among metastatic cells. This does not imply identical biology across sites but indicates shared transcriptional programs permissive for dissemination/survival.

### 4.3. A putative transition state and its functional features

Between normal and malignant epithelium we identified a putative transition phenotype. Compared with normal cells, transition cells showed attenuation of lineage functions (e.g., reduced gastric-acid-related signatures) and enrichment of stress-adaptation programs. Along pseudotime, normal cells occupied early positions, transition cells expanded, and a bifurcation led toward tumor-like states. We emphasize that pseudotime reflects relative transcriptional progression rather than calendar time; therefore, statements about temporal precedence are provisional. The observation that a metastasis-like branch appears early in pseudotime should be interpreted as consistent with earlier activation of dissemination-related programs, not as proof that metastatic tumors chronologically precede primary tumors.

### 4.4. Copper-linked stress programs along the progression axis

GO/KEGG enrichment of pseudotime-associated genes highlighted themes expected under copper handling and cytotoxic stress (including redox/NRF2, ER stress/UPR, p53-linked DNA-damage and apoptosis, and mitochondrial apoptotic processes) together with epithelial junction/ECM remodeling, migration, hypoxia/angiogenesis, and inflammatory signaling. Single-cell GSVA/GSEA/module scoring further supported these signals: stress response to copper ion increased from normal to transition epithelial cells and decreased along the tumor branch, indicating maximal copper-linked stress in the transitional state. In contrast, metastatic epithelial cells exhibited attenuated copper-stress signatures with enhanced motility/tumor programs. These patterns are compatible with a model in which copper-related stress accompanies early epithelial remodeling; they do not, by themselves, establish causality.

### 4.5. Immune-related changes and putative evasion

Primary tumor epithelial cells displayed reinforced cell-cycle/proliferation programs and attenuated immune-responsive pathways, with dysregulation of checkpoint-related genes detectable already in transition cells. TIDE-based assessments suggested greater immune dysfunction rather than exclusion, consistent with interference in effector function despite infiltration. The intersection of metabolic stress and immune modulation is therefore a plausible, testable axis in early transformation.

### 4.6. Genetic evidence consistent with a causal contribution of copper

Two-sample MR using genetically proxied circulating copper as exposure and benign gastric tumors (FinnGen) and gastric cancer (GWAS Catalog) as outcomes yielded 6 linkage disequilibrium-independent instruments after clumping. Instrument strength appeared acceptable (per-SNP *F* > 10); we evaluated I²_GX for potential regression dilution of MR-Egger and conducted Cochran *Q*, MR-Egger intercept, and MR-PRESSO (global/outlier/distortion) diagnostics. Across estimators (IVW, weighted median, mode-based), effect directions were concordant; scatter/funnel and leave-one-out analyses indicated no dominant single-variant influence. Taken together, the MR results are consistent with higher genetically proxied circulating copper being deleterious for benign and malignant gastric tumor risk under standard MR assumptions. Nonetheless, with n = 6 instruments, residual pleiotropy, weak-instrument bias, and locus-specific mechanisms cannot be excluded; thus, MR findings do not constitute proof of causality.

### 4.7. Limitations

Pseudotime inference is sensitive to parameterization and gene-set choice; our ordering genes were epithelial subcluster markers and the single branch point was data-driven, so interpretations are conservative. The transition phenotype is bioinformatically defined and requires orthogonal validation in tissue. MR estimates reflect systemic, genetically proxied copper and remain susceptible to pleiotropy, limited instrument number, and confounding by copper-related disease states; dietary copper and tissue-specific biology are not isolated.

### 4.8. Working model

Integrating single-cell trends with MR, copper-linked metabolic stress appears most pronounced in the transitional epithelial state, while metastasis-bound cells show attenuated copper-stress signatures and enhanced motility/tumor programs; these patterns are consistent with, but do not prove, a deleterious contribution of higher systemic copper to gastric tumor risk.

## 5. Conclusions

Across publicly available single-cell transcriptomes from primary and metastatic gastric cancer, a reproducible three-state landscape emerges.

1. A *primary-tumor phenotype* marked by proliferative programs and immune-evasion features.
2. A *metastatic phenotype* characterized by attenuated copper-toxicity metabolism and heightened migratory potential.
3. An *intermediate (transition) phenotype* with pronounced copper-stress/toxicity signatures that are antithetical to the metastatic state.

**Figure 7.**
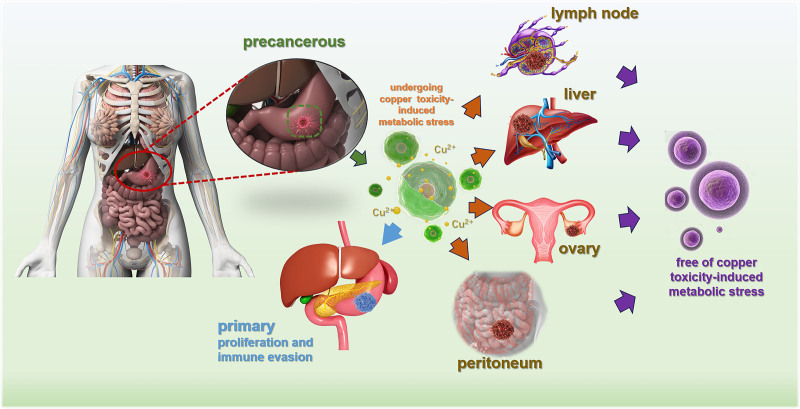
Illustrative summary diagram.

Trajectory analyses place the transition phenotype between primary and metastatic states, with copper-stress programs peaking along the putative route to dissemination and diminishing in the metastatic endpoint. This pattern supports a model (Fig. 7) in which selection for reduced copper-toxicity responses accompanies acquisition of migratory traits. These findings are derived from integrative bioinformatic analyses and should be interpreted as hypothesis-generating rather than causal. They are consistent with (but do not by themselves prove) an adverse contribution of systemic copper biology to gastric tumor progression, and they do not isolate effects related to dietary exposure, tissue-specific contexts, or co-existing copper-related disease states. The model offers testable predictions for orthogonal validation.

## Author contributions

**Conceptualization:** Kewei Bi, Xuge Wei, Fu Ren.

**Methodology:** Kewei Bi, Xuge Wei.

**Data curation:** Kewei Bi.

**Formal analysis:** Xuge Wei, Chao Han, Kewei Bi.

**Funding acquisition:** Fu Ren.

**Investigation:** Xuge Wei, Fu Ren, Kewei Bi.

**Methodology:** Kewei Bi.

**Project administration:** Kewei Bi, Fu Ren.

**Supervision:** Fu Ren, Kewei Bi.

**Validation:** Chao Han.

**Visualization:** Xuge Wei, Chao Han, Kewei Bi.

**Writing – original draft:** Xuge Wei, Chao Han, Kewei Bi.

**Writing – review & editing:** Ning Li, Baishi Wang.

## Supplementary Material






