# Supplementary material for: Copper-linked metabolic stress as a potential contributor to gastric epithelial transformation and metastasis
Source: Medicine (Baltimore). 2025 Dec 26;104(52):e46590. doi: 10.1097/MD.0000000000046590 (PMC12747020; doi:10.1097/MD.0000000000046590)

**SUPPLEMENTARY FIGURE 1. PCA before and after batch-effect correction using CCA.**
(**A**, **C**, **E**, **G**) PCA of the uncorrected data; (**B**, **D**, **F**, **H**) PCA after batch-effect correction via Seurat CCA–based integration.


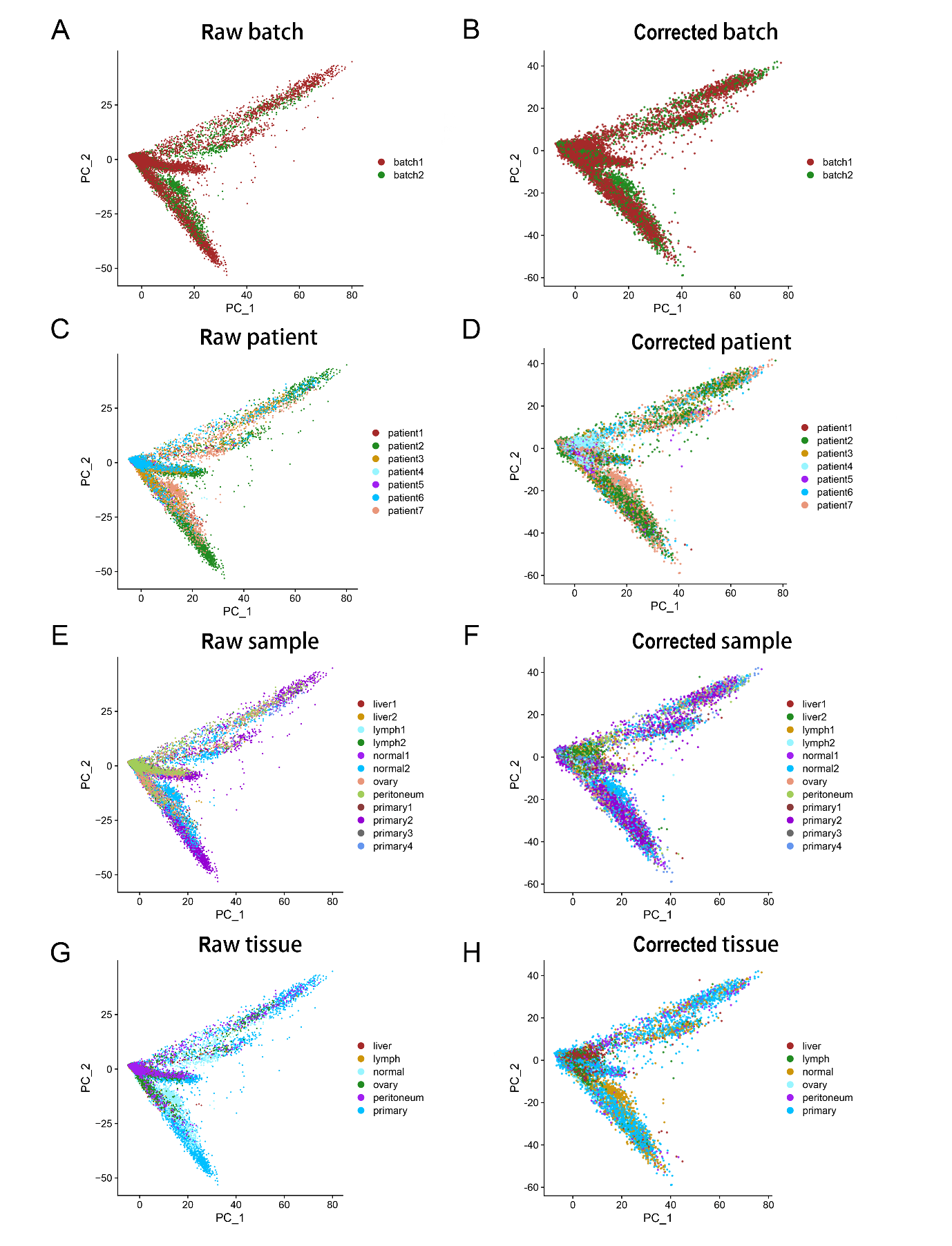


**SUPPLEMENTARY FIGURE 2. The expression profile of genes characteristic to specific cell types.**

**(A-I)** Genes showing characteristic expression patterns in specific cell types.


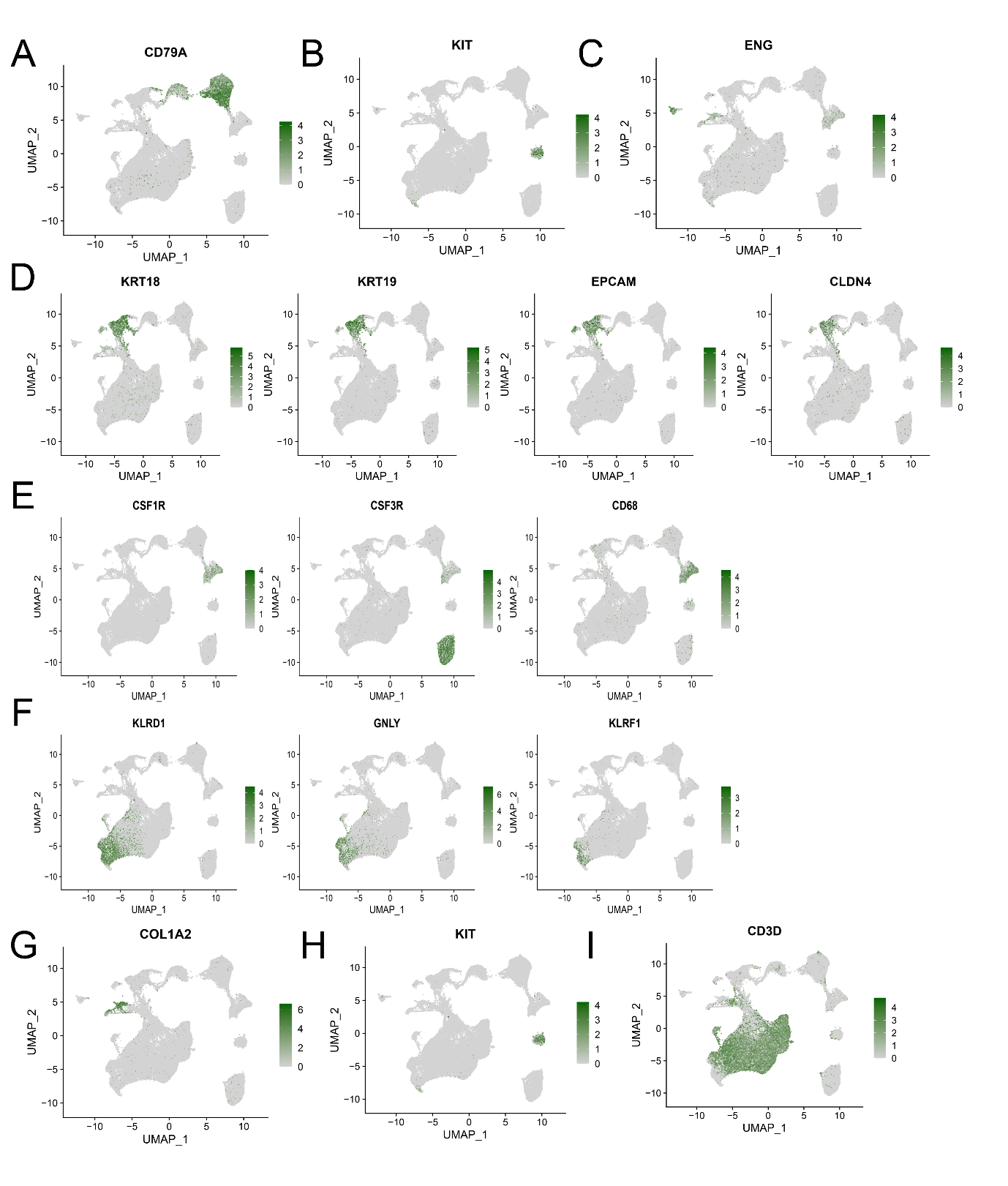


**SUPPLEMENTARY FIGURE 3. The tissue origins of different epithelial cell subgroups and the expression of characteristic marker genes in malignant epithelial tumor cells.**

**(A)** Highlighting the tissue origin of cells within the epithelial cell clusters. (**B**) The expression of marker genes characteristic of malignant epithelial cells.


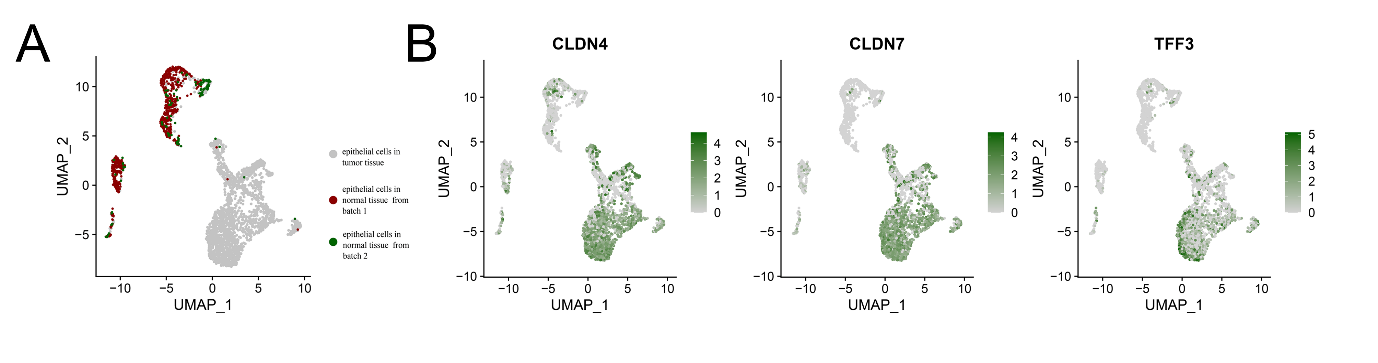


**SUPPLEMENTARY FIGURE 4. Copper-stress activity along the epithelial pseudotime trajectory.**
Per-cell module scores for the GO term “stress response to copper ion” were computed and mapped onto the epithelial pseudotime trajectory.


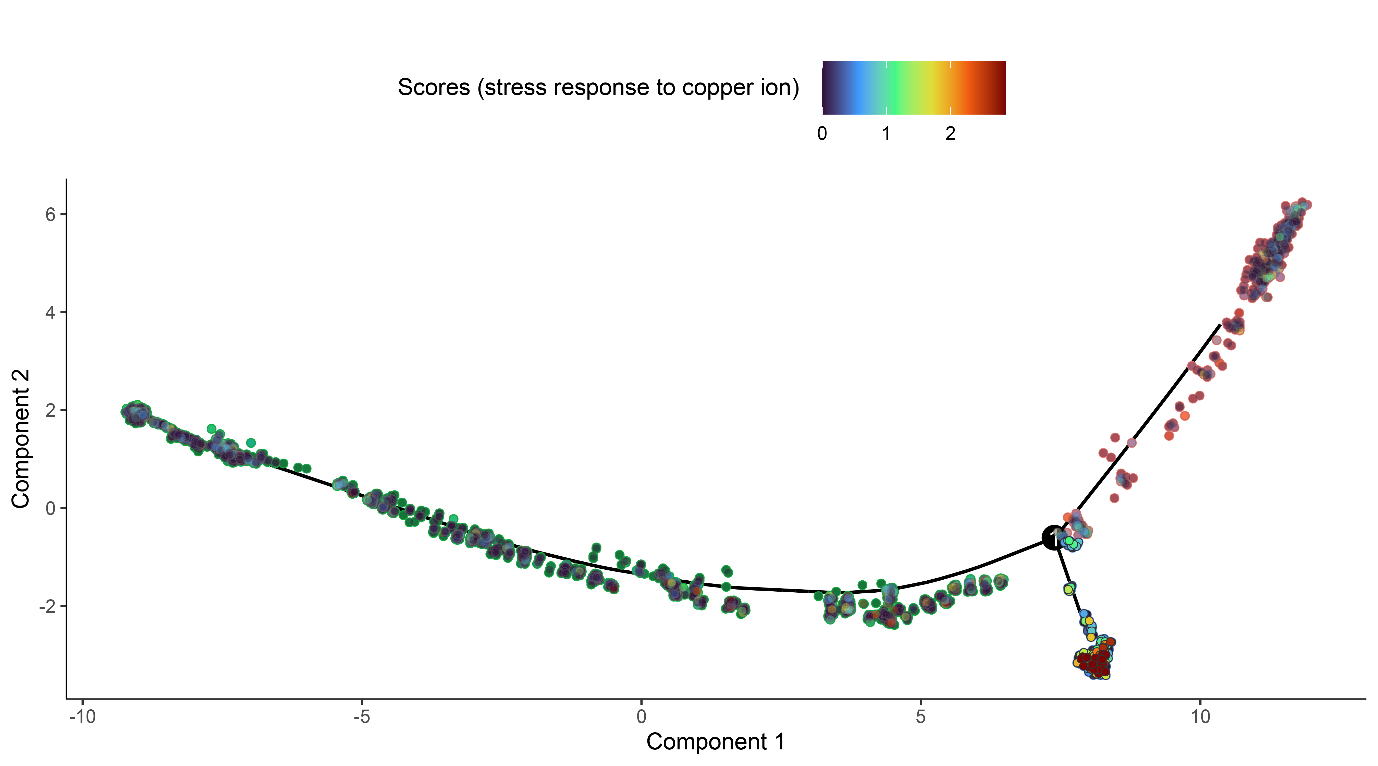


**SUPPLEMENTARY FIGURE 5. Forest plot and funnel plot presenting the related test effects of instrumental variable SNPs.**

**(A**-**B)** The forest plot shows the individual and combined effects of instrumental variable SNPs on benign and malignant gastric tumors, respectively. (**C**-**D**) Funnel plots displaying the heterogeneity among the instrumental variable SNPs in benign and malignant tumors, respectively. (**E**-**F**) Forest plots showing the results of sensitivity tests for the instrumental variable SNPs in benign and malignant tumors, respectively.


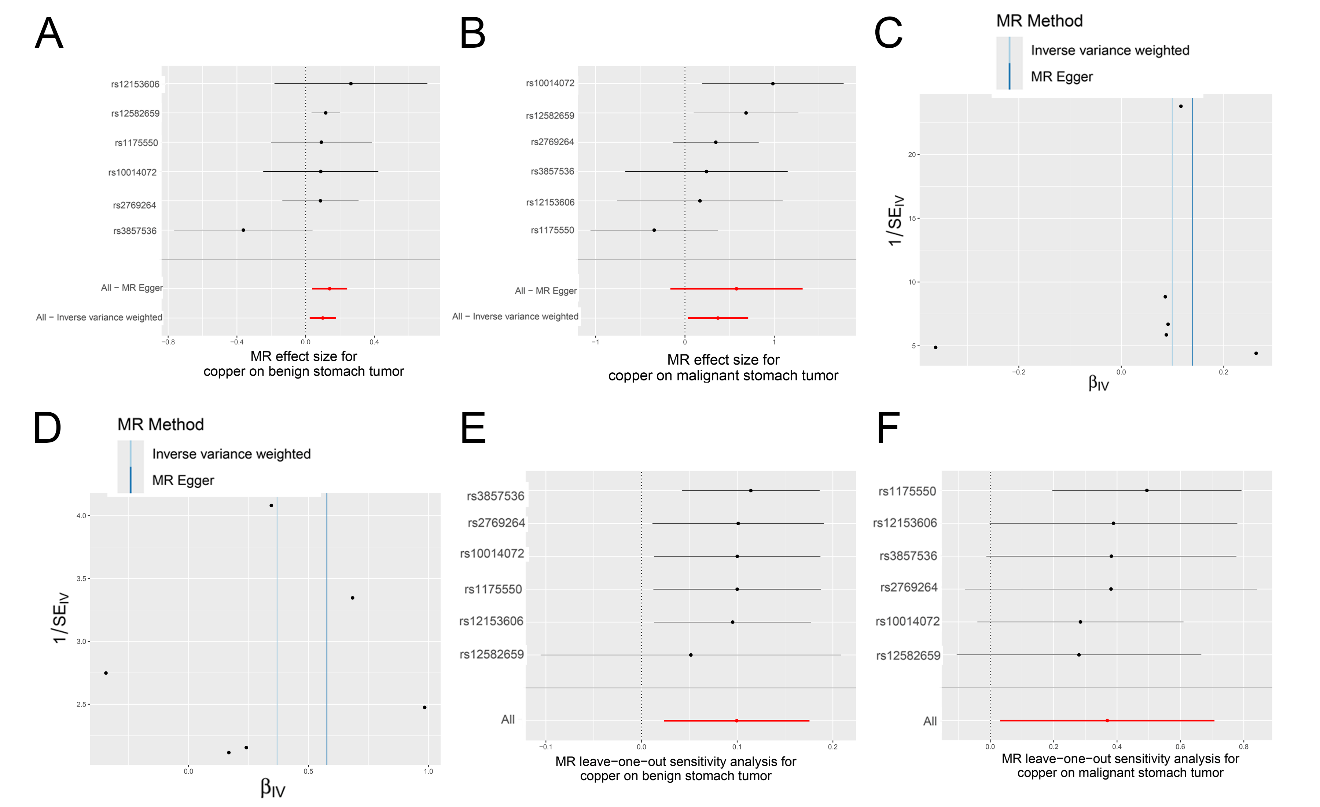

Supplement: Supplementary file 2 [file medi-104-e46590-s002.docx]
